# Peripheral blood circular RNA hsa_circ_0124644 can be used as a diagnostic biomarker of coronary artery disease

**DOI:** 10.1038/srep39918

**Published:** 2017-01-03

**Authors:** Zhenzhou Zhao, Xuejie Li, Chuanyu Gao, Dongdong Jian, Peiyuan Hao, Lixin Rao, Muwei Li

**Affiliations:** 1 Department of Cardiology, People’s Hospital of Zhengzhou University, Zhengzhou University, Zhengzhou, China; 2 Department of Cardiology, The First Affiliated Hospital of Zhejiang University, Zhejiang University, Hangzhou, China

## Abstract

The aim of the present study was to investigate the expression of circular RNAs (circRNAs) in the peripheral blood of coronary artery disease (CAD) patients and the potential use of circRNAs as diagnostic biomarkers of CAD. We first analysed peripheral blood circRNAs of 12 CAD patients and 12 control individuals by RNA microarray and found that 22 circRNAs were differentially expressed between these two groups: 12 were upregulated, and 10 were downregulated. Then, we selected 5 circRNAs as candidate biomarkers under stricter screening criteria and verified them in another group of subjects consisting of 30 control individuals and 30 CAD patients with different SYNTAX scores. These 5 circRNAs were all remarkably increased in the CAD group. Hsa_circ_0124644 had the largest area under the curve (AUC). We tested hsa_circ_0124644 in an independent cohort consisting of 115 control individuals and 137 CAD patients. After we included the risk factors for CAD, the AUC slightly increased from 0.769 (95% confidence interval = [0.710–0.827], *P* < 0.001) to 0.804 ([0.751–0.857], *P* < 0.001), and when combined with hsa_circ_0098964, the diagnostic value slightly increased. Taken together, our results suggest that hsa_circ_0124644 can be used as a diagnostic biomarker of CAD.

According to a 2014 statistical summary by the World Health Organization (WHO), cardiovascular diseases are the most lethal non-communicable diseases (NCD), accounting for 46% of all NCD mortality. Due to the ageing of the Chinese population, the death toll from cardiovascular disease is rapidly rising^1^. The high morbidity and mortality of coronary artery disease (CAD) is a substantial threat to human health and a heavy burden to social economies worldwide. Despite the current standardized treatments of CAD, which include medications, percutaneous coronary intervention (PCI) and coronary artery bypass graft surgery (CABG), the prognosis remains unsatisfactory in some patients^2^,^3^. This discrepancy is mainly because the current diagnostic methods cannot simultaneously achieve high sensitivity and convenience, which obviously increases the missed diagnosis rate. Therefore, a new, highly sensitive and convenient diagnostic biomarker of CAD will be highly valuable.

Non-coding RNAs have been considered the “dark matter” of the genome due to their great diversity and unclear functions. However, recent studies have revealed that though non-coding RNAs do not code for proteins directly, they do play a regulatory role in the transcription and translation of protein-coding genes^4^. Circular RNAs (circRNAs) are derived from the reverse splicing of exons, introns, or both to form a closed continuous loop^5^,^6^. Because circRNAs do not have 5′ or 3′ ends, they are free of exonuclease-mediated degradation and more stable than most linear RNAs^7^. CircRNAs regulate gene expression through multiple mechanisms^8^. They may act as “miRNA sponges”, thus participating in posttranscriptional regulation through competitively binding miRNA^9^, interacting with snRNA or RNA polymerase II^10^ combining with transcription factors, or competitively regulating classic RNA splicing^11^. CircRNA expression is tissue-specific and time-specific. For example, ciRS-7 is highly expressed in brain tissue and expressed at low levels in other tissues^8^. At different stages of biological development, the types and expression levels of circRNAs also vary significantly^12^,^13^. Substantial amounts of circRNAs are widely distributed in the cytoplasm, the nucleus and a variety of body fluids, including saliva and serum exosomes^14^,^15^.

Studies have shown that circRNAs are intimately involved in cancer, cardiac hypertrophy-induced heart failure, pulmonary fibrosis, myotonic dystrophy, Alzheimer’s disease and other diseases^11^,^16^,^17^,^18^. Li *et al*.^19^ found that the expression of hsa_circ_002059 in gastric cancer tissue is significantly higher compared with that in adjacent tissues; this circRNA is now used in the clinic as a novel biomarker of gastric cancer. Li *et al*.^20^ demonstrated that circ-ITCH can be used to diagnose oesophageal cancer, whereas Qin *et al*.^21^ identified hsa_circ_0005075 as a potential biomarker of hepatocellular carcinoma. For cardiovascular disease, a genome-wide association study (GWAS) revealed a correlation between SNPs on chromosome 9p21.3 and susceptibility to atherosclerosis^22^. Chromosome 9p21.3 includes the INK4/ARF gene, antisense noncoding RNA sequence (ANRIL), and the MTAP gene. C.E e*t al.*^17^ found that the circular isoform of ANRIL (cANRIL) may affect the progression of CAD by regulating INK4/ARF expression.

In this study, we compared the peripheral blood circRNA profiles between CAD patients and matched control subjects by microarray analysis and then verified our findings in larger independent cohorts. Our results indicated that hsa_circ_0124644 may be a sensitive and specific biomarker for diagnosing CAD.

## Methods

### Study cohorts

Three hundred thirty-six participants were included in this study, consisting of a test group of outpatients and inpatients from the cardiovascular department of People’s Hospital of Zhengzhou University from July 2015 to June 2016, and a control group of subjects was recruited from the cardiovascular department or physical examination department. The participants in this study were divided into three cohorts (the clinical and demographic characteristics are summarized in Supplementary Tables S1–S3). Subjects were excluded according to the following criteria: 1) malignancies; 2) liver and kidney dysfunction; 3) any other clinically systemic acute or chronic inflammatory diseases; 4) history of acute myocardial infarction (AMI), PCI, or CABG; 5) autoimmune disease; 6) uncontrolled hypertension; or 7) malignant arrhythmias or valvular heart disease. This study was approved by the Ethics Committee of the People’s Hospital of Zhengzhou University. All selected patients provided informed consent, and the study protocol conformed to the ethical guidelines of the Declaration of Helsinki (1975).

### Study design

**Figure 1:**
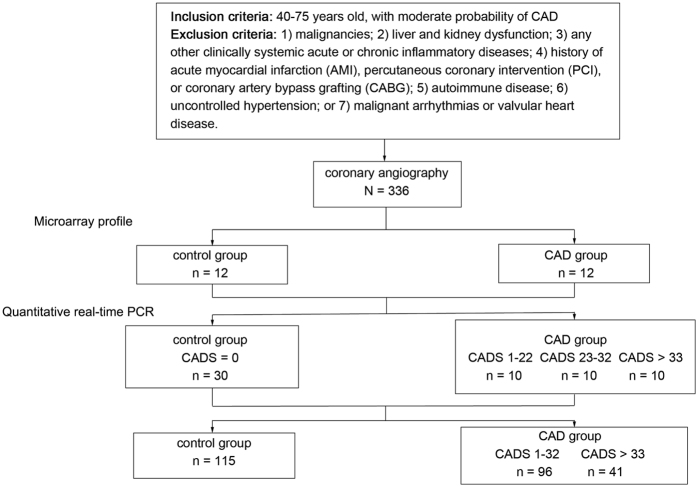
Study flow chart.

The flowchart for this study is shown in Fig. 1. All participants were examined by coronary angiography (CAG) to verify the presence of CAD. We first selected 12 CAD patients and 12 clinically matched control subjects and isolated total RNAs from their venous blood samples for microarray analysis. The selected circRNAs were then verified in the second cohort, which contained 30 control subjects and 30 patients with different severities of CAD (as measured by the SYNTAX score^23^ and expressed as the CADS). The correlations between the expression level of a particular circRNA and the CADS were investigated. Finally, the circRNA with the best performance was chosen as the biomarker of CAD, and its diagnostic value was tested in a third cohort (control group n = 115; CAD group n = 137).

### Definition of CAD and collection of blood samples

CAD was diagnosed by CAG and defined as coronary stenosis ≥50% in at least one of the coronary arteries according to the American College of Cardiology/American Heart Association guidelines^24^. The diagnosis was made independently by two experienced interventional cardiology physicians through visual observation. The control individuals showed no signs of coronary atherosclerosis or microvascular diseases as indicated by negative treadmill exercise testing (TET) and emission computed tomography (ECT) results.

Blood samples (2 ml) were collected from the median cubital vein with an ethylenediaminetetraacetic acid (EDTA) anticoagulated vacutainer, and total RNA was extracted immediately.

### Extraction of total RNA and quantitative polymerase chain reaction (qPCR)

Within 20 min of blood collection (the blood samples were stored in an ice box at 4 °C during this period); total RNA was extracted from 1 ml whole blood with a rapid blood total RNA extraction kit (Biotech, China) according to the manufacturer’s instructions. The extracted RNA was dissolved in RNase-free water and measured using a Nanodrop 2000 (Thermo Scientific, USA). The integrity of the RNA was determined by 1% formaldehyde denaturing gel electrophoresis. The samples were used in the reverse transcription reactions when the A260/A280 was between 1.8 and 2.0 and the concentration >200 ng/μl. The reverse transcription of quantified RNA was performed using a PrimeScript RT reagent kit (Takara Bio, Japan) according to the manufacturer’s instructions. Briefly, 500 ng RNA and 2 μl PrimeScript RT Master Mix (Takara Bio, Japan) was mixed with RNase-Free water to a final volume of 10 μl. Then, the mixture was incubated in a water bath at 37 °C for 15 min to complete the reverse transcription and acquire total cDNA. QPCR was performed with 5 ng cDNA, 5 μl SYBR-Green Premix Ex Taq (Takara Bio, Japan), 3 μl RNase-free water and 1 μl primers (reverse and forward) and monitored using an ABI PRISM 7500 sequence detection system (applied Biosystems, Life Technologies, USA) at 95 °C for 3 min and amplified by 40 cycles of denaturing at 95 °C for 10 s and 60 °C for 30 s. The C_T_ value was the fractional cycle number at which the fluorescence exceeded the given threshold. GAPDH was used to normalize the RNA preparation. The relative expression levels of circRNAs were determined using the 2^−△CT^ method. The primers used for qPCR are listed in Supplementary Table S4.

### CircRNA microarray expression profiling

The peripheral blood total RNAs extracted from the subjects (12 in the CAD group, 12 in the control group) were used for microarray analysis. The purity and concentration of RNA were determined from the OD260/280 readings using a spectrophotometer (NanoDrop ND-1000). The RNA integrity was determined using a Bioanalyzer 2100 (Agilent Technologies, USA). RNA digestion, amplification and labelling were performed according to the manufacturer’s protocol. The labelled RNAs were hybridized onto the microarray (Human circRNA array, version 2.0) after purification. Each array contained probes interrogating approximately 170,340 human circRNAs. The circRNA array data were analysed using GeneSpring software V13.0 (Agilent). To select the differentially expressed circRNAs, we used threshold values of ≥2 and ≤−2-fold change and a *t*-test *P*-value of 0.05.

### Statistical analysis

The data were presented as the means ± standard deviations, medians (quartiles), or proportions when appropriate. In the scatter plot depicting the circRNA expression, the horizontal lines represent the medians. Categorical variables were tested using the Chi-square test, and continuous variables were first tested by Kolmogorov-Smirnov and Shapiro-Wilk tests to verify whether the data sets were normally distributed. If normally distributed, the data were analysed using two-tailed Student’s *t*-tests or, if non-normally distributed, using Mann-Whitney *U* tests. The clinical diagnostic value of a given circRNA was verified by receiver operating characteristic (ROC) curve analysis in which an area under the curve (AUC) = 0.5 indicated no diagnostic value. The cut-off value and corresponding sensitivity and specificity could be identified through ROC curve analysis. The correlation between the circRNA expression level and the CADS was evaluated by Pearson’s correlation test. To calculate the odds ratio (OR), the relative expression of the analysed circRNAs were multiplied by 10 and logistic regression analyses were performed. The crude OR was adjusted after introducing risk factors for CAD, including smoking, hypertension, diabetes mellitus (DM), low density lipoprotein (LDL) and total cholesterol (TC). *P* < 0.05 was considered statistically significant. All statistical analyses were performed using SPSS 22.0 (SPSS Inc., Chicago, IL, USA).

## Results

### Peripheral blood circRNA profile in CAD patients

**Figure 2:**
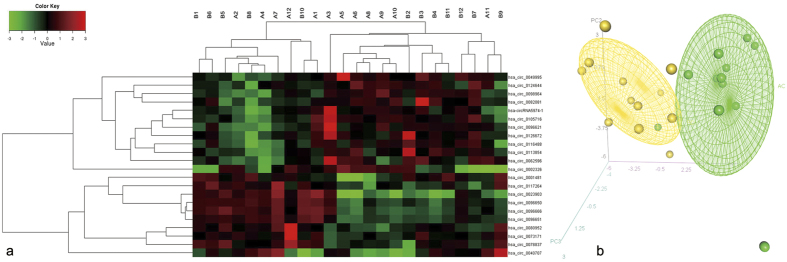
Heat map and PCA analysis of circRNA microarray profile in control individuals (n = 12) and CAD patients (n = 12). (**a**) Heat map. The expression of circRNAs is hierarchically clustered on the y-axis; corresponding circRNAs are shown on the right. Blood samples from control individuals and CAD patients are hierarchically clustered on the x-axis. Expression values are presented in red and green to indicate upregulation and downregulation, respectively. Numbers with A indicate control individuals, and numbers with B indicate CAD patients. (**b**) PCA analysis.

To identify whether circRNAs are differentially expressed between CAD patients and normal subjects, we extracted peripheral blood total RNA from 12 CAD patients and 12 controls for circRNA microarray assay analysis. The levels of circulating circRNAs varied significantly between the CAD and control groups (Fig. 2). Twenty-two circRNAs were differentially expressed between the two groups, of which 12 circRNAs were upregulated and 10 were downregulated in CAD patients (the fold changes are shown in Supplementary Table S5). To identify the most clinically applicable biomarker, we chose the 5 circRNAs that showed the highest fold changes (*P* < 0.01) from the 12 upregulated circRNAs for further analysis: hsa_circ_0082081, hsa_circ_0113854, hsa_circ_0124644, hsa_circ_0098964 and hsa-circRNA5974-1 (highlighted in Supplementary Table S5).

### Verification of the circRNA profile by qPCR

**Figure 3:**
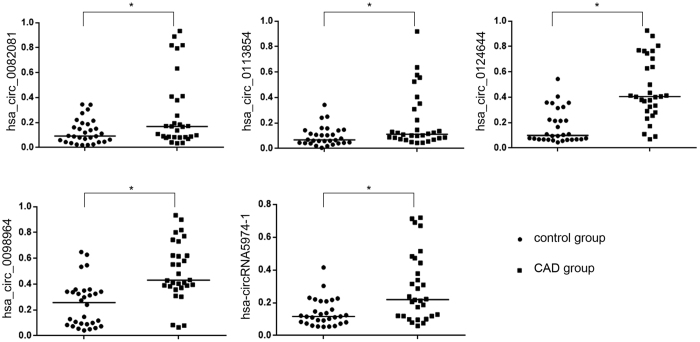
Expression levels of circRNAs quantified by qPCR. The cohort included 30 control individuals and 30 CAD patients with different CADS. The relative expression levels of circRNAs were normalized to levels of the control (hGAPDH). *P < 0.05 compared to the control group.

We verified the 5 candidate biomarkers by qPCR in another independent cohort consisting of 30 control subjects (CADS = 0, n = 30) and 30 CAD patients with different CADS (CADS 1–22, n = 10; CADS 23–32, n = 10; CADS > 33, n = 10). The results confirmed the findings of the microarray analysis (Fig. 3), as the levels of these 5 circRNAs were significantly increased in the CAD patients; there was a 2.7-fold change in the expression of hsa_circ_0124644, and 2.2-, 2.1-, 2.0-, and 2.1-fold changes in the expression levels of hsa_circ_0082081, hsa_circ_0113854, hsa_circ_0098964 and hsa-circRNA5974-1, respectively.

### ROC curve analysis of the differentially expressed circRNAs

**Figure 4:**
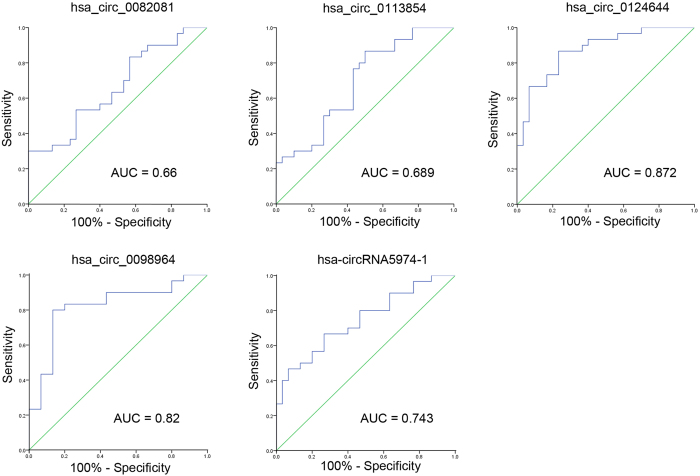
ROC curve analyses of circRNAs. The AUC values are given on the graphs.

To compare the diagnostic value of these 5 circRNAs as candidate biomarkers of CAD, we performed ROC curve analysis for each circRNA. As shown in Fig. 4, the AUC was larger than 0.500 for all 5 candidate circRNAs, suggesting their potential diagnostic value. Notably, the AUC of hsa_circ_0124644 reached 0.872 ([0.785–0.960], *P* < 0.001), which was the largest among the 5 circRNAs. The AUC of the other circRNAs were 0.660 ([0.522–0.798], *P* = 0.033) for hsa_circ_0082081, 0.689 ([0.555–0.823], *P* = 0.011) for hsa_circ_0113854, 0.820 ([0.707–0.933], *P* < 0.001) for hsa_circ_0098964 and 0.743 ([0.619–0.867], *P* = 0.001) for hsa-circRNA5974-1. The sensitivity and specificity of each circRNA were determined based on the cut-off value (shown in Table 1), and hsa_circ_0124644 showed the highest sensitivity and specificity (86.7% and 76.7%).

### The correlation between the expression levels of circRNAs and CADS

**Figure 5:**
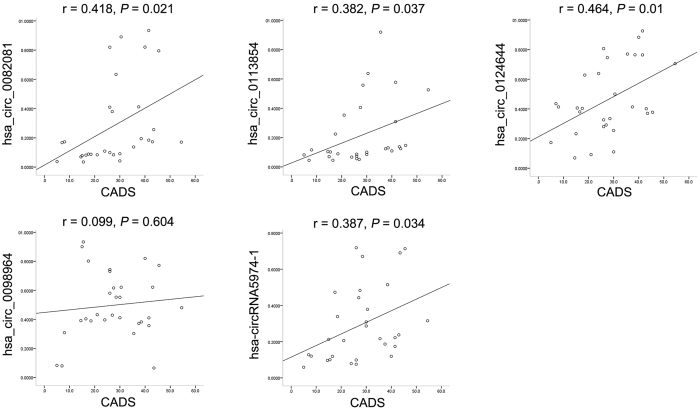
Pearson correlation coefficients between circRNAs and CADS.

In clinical practice, the severity of CAD is often measured by the SYNTAX score, which guides the optimal intervention. In this study, we examined the correlation between circRNA levels and the CADS by Pearson’s correlation test and found that 4 of the 5 circRNAs were correlated with the CADS (Fig. 5). Among these circRNAs, hsa_circ_0082081 and hsa_circ_0124644 were moderately correlated with the CADS, hsa_circ_0113854 and hsa-circRNA5974-1 were weakly correlated with the CADS and hsa_circ_0098964 was not correlated with the CADS. Based on the AUCs of the 5 candidates and their correlations with the CADS, we chose hsa_circ_0124644 as a potential biomarker for the diagnosis of CAD.

### Clinical verification of the biomarker

**Figure 6:**
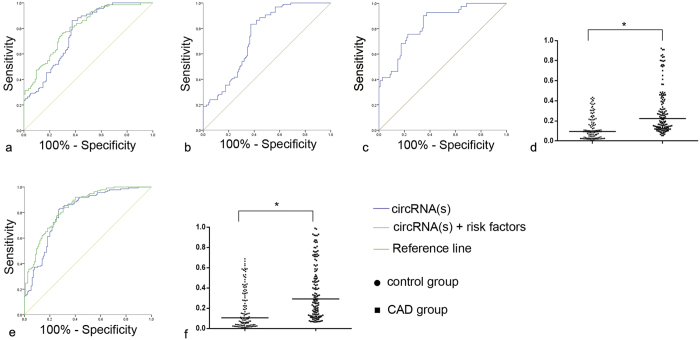
The expression levels of hsa_circ_0124644 and hsa_circ_0124644 combined with hsa_circ_0098964 in CAD patients and controls. Control group, n = 115; CAD group, n = 137. a: ROC curve analyses of hsa_circ_0124644 in CAD patients and controls; b and c: ROC curve analyses of hsa_circ_0124644 in low and moderate CADS and high CADS patients, respectively; d: The expression levels of hsa_circ_0124644; e: ROC curve analyses of hsa_circ_0124644 combined with hsa_circ_0098964 in CAD patients and controls; f: The expression levels of hsa_circ_0124644 combined with hsa_circ_0098964. *: *P* < 0.05 compared to the control group.

To evaluate the actual diagnostic value of hsa_circ_0124644 in clinical settings, we tested this circRNA in another independent cohort consisting of 137 CAD patients and 115 control subjects. As shown in Fig. 6, hsa_circ_0124644 was significantly upregulated in the CAD group, with a 2.2-fold change. ROC curve analysis demonstrated an AUC of 0.769 ([0.710–0.827], *P* < 0.001), and its sensitivity and specificity were 0.861 and 0.626, respectively. The crude OR was 1.856 ([1.494–2.306], *P* < 0.001). After including the risk factors for CAD (smoking, hypertension, DM, LDL and TC), the AUC slightly increased to 0.804 ([0.751–0.857], *P* < 0.001), with a sensitivity of 0.759, a specificity of 0.704 and an adjusted OR of 1.931 ([1.511–2.467], *P* < 0.001). These results suggested that hsa_circ_0124644 may be a sensitive and specific biomarker of CAD. To investigate the diagnostic value of hsa_circ_0124644 for low and moderate CADS and high CADS, we distributed the third cohort into two groups (CADS: 1–32, n = 96; CADS > 33, n = 41) according to the CADS. In the group with low and moderate CADS, the ROC analysis demonstrated an AUC of 0.740 ([0.674–0.806], *P* < 0.001), and its sensitivity and specificity were 0.833 and 0.626, respectively. In the group with high CADS, the AUC, sensitivity and specificity were 0.836 ([0.768–0.904], *P* < 0.001), 0.902 and 0.652, respectively. The AUC of the high CADS group was higher than that of the low and moderate CADS group, which were both larger than 0.700; this result implied that the diagnostic value of hsa_circ_0124644 was appropriate at all CADS values, though it was more accurate in the higher CADS group.

### Hsa_circ_0124644 combined with hsa_circ_0098964

To improve the diagnostic value of the biomarker, we introduced hsa_circ_0098964. As shown in Fig. 6, hsa_circ_0098964 was significantly upregulated in the CAD group and showed a 1.9-fold change. We combined hsa_circ_0098964 with the previously tested circRNA and tested this combination as a new biomarker using ROC analysis. The results showed that the AUC was 0.811 ([0.756–0.865], *P* < 0.001), the sensitivity was 0.825 and the specificity was 0.730. After introducing the risk factors for CAD (smoking, hypertension, DM, LDL and TC), the AUC slightly increased to 0.843 ([0.796–0.891], *P* < 0.001), the sensitivity was 0.832 and the specificity was 0.696. These results implied that, compared with hsa_circ_0124644 alone, the combination of hsa_circ_0124644 and hsa_circ_0098964 as a biomarker has a higher diagnostic value for CAD.

## Discussion

CAD has high morbidity and mortality worldwide and places a huge burden on social economies due largely to the difficulties of obtaining an early diagnosis. Currently, CAD can be diagnosed by either non-invasive or invasive methods. The non-invasive methods include electrocardiogram (ECG), TET, Holter monitoring and coronary computed tomography angiography (CTA), which each have their limitations. ECG shows poor sensitivity and specificity. Holter monitoring can diagnosis CAD only when it captures the dynamic changes during the onset of angina. TET is not suitable for some elderly patients and those with limited physical activity, and some patients may experience rupture of an unstable plaque or the occurrence of adverse cardiac events during the induction of myocardial ischaemia. Finally, CTA is somewhat expensive. Invasive examinations mainly include CAG and intravascular ultrasound (IVUS). CAG is the gold standard for the diagnosis of CAD, and IVUS, in which a miniature ultrasound probe is sent into the vessel lumen to obtain a tomographic scan, can accurately and intuitively determine residual stenosis and guide stenting^25^. In China, however, many low-income families cannot afford these examinations, and many patients with mild symptoms and conservative ideas are not willing to undergo such invasive examinations. Therefore, a diagnostic method that is both low-cost and highly sensitive and specific is urgently needed to facilitate the diagnosis of CAD.

CircRNAs are a special class of non-coding RNAs that are abundant in body fluids. Some lncRNAs and miRNAs have been shown to be closely related to CAD^26^,^27^,^28^. Therefore, we speculated that circRNAs may also be potential biomarkers of CAD. Due to their circular structure and resistance to RNA exonuclease, circRNAs are much more stable than linear RNA. Moreover, in some tissues, the expression levels of circRNAs are 10 times higher than the expression levels of linear RNAs, which also makes circRNA a better biomarker^7^.

In this study, we first explored differentially expressed circRNAs in CAD patients and control individuals, and 5 significant differentially expressed circRNAs were selected for verification. Then, hsa_circ_0124644 was selected and further tested in larger cohorts to demonstrate its high diagnostic value as a biomarker of CAD. Compared with hsa_circ_0124644, the combined use of hsa_circ_0124644 and hsa_circ_0098964 as a biomarker increased the AUC from 0.769 ([0.710–0.827], *P* < 0.001) to 0.808 ([0.756–0.860], *P* < 0.001), which indicated an improvement in the diagnostic value. Moreover, the Pearson’s correlation test demonstrated that the expression of hsa_circ_0124644 was moderately correlated with the CADS, implying that hsa_circ_0124644 might be deeply involved in the pathologies of CAD. Therefore, this circRNA can reflect the severity of CAD and have great diagnostic value for CAD.

The field of circRNAs is quite a new area, and we have not yet found any definite evidence to demonstrate the functions of hsa_circ_0124644. The result of gene ontology enrichment analysis suggests that this circRNA is strongly correlated with cell apoptosis, the Robo receptor signalling pathway and some other cellular processes. Moreover, hsa_circ_0124644 is closely related to intercellular adhesion factors. It is well known that these processes all play important roles in the progression of CAD^29^,^30^,^31^,^32^,^33^,^34^,^35^. Therefore, we speculate that hsa_circ_0124644 may be involved in the progression of CAD through these biological processes.

In addition to CAG, many methods are currently used to diagnose CAD, including routine ECG, Holter monitoring, TET and CTA. The sensitivity and specificity of routine ECG have been shown to be 0.290 and 0.670^36^. The sensitivity and specificity of Holter monitoring are 0.649 and 0.894^37^. The sensitivity and specificity of TET are 0.731 and 0.693^38^. Finally, the sensitivity and specificity of CTA are 0.920 and 0.750^39^. We found that the sensitivity and specificity of hsa_circ_0124644 were 0.861 and 0.626, respectively. When hsa_circ_0124644 was combined with hsa_circ_0098964, the sensitivity and specificity of the combined biomarker were 0.825 and 0.730, respectively. Based on these comparisons, we think that the diagnostic value of the hsa_circ_0124644 and the combination of hsa_circ_0124644 and hsa_circ_0098964 are greater than that of routine ECG and TET, which are approximately equal to Holter monitoring and significantly lower than CTA in terms of sensitivity and specificity. However, when considering the cost and convenience of diagnostic methods, we believe that our biomarker will significantly improve the diagnosis of CAD.

Our study was the first to investigate the circRNA profile in the whole blood of CAD patients. However, as a single-centre study, the subjects were geographically concentrated, and whether such circRNA profile can also be detected in other regions or countries is in doubt. The conclusions of this study require further verification in larger and more diverse cohorts. In addition, only hsa_circ_0124644 was validated in this study, and the expression levels of other circRNAs remain to be explored.

In conclusion, this study is the first to investigate the circRNA profile in the peripheral blood of CAD patients, to determine its correlation with the severity of CAD, and to test the potential of circRNA as a diagnostic biomarker of CAD. The biomarker identified in this study (hsa_circ_0124644) can be easily tested using peripheral blood at a relatively low cost, yet the specificity and sensitivity are relatively high, and the diagnostic value would slightly increase after introducing hsa_circ_0098964, making hsa_circ_0124644 a powerful tool in the diagnosis of CAD.

## Additional Information

**How to cite this article**: Zhao, Z. *et al*. Peripheral blood circular RNA hsa_circ_0124644 can be used as a diagnostic biomarker of coronary artery disease. *Sci. Rep.* **7**, 39918; doi: 10.1038/srep39918 (2017).

**Publisher's note:** Springer Nature remains neutral with regard to jurisdictional claims in published maps and institutional affiliations.

## Supplementary Material

Supplementary Tables

## Figures and Tables

**Table 1:** Sensitivity and specificity of the candidate biomarkers in controls and CAD patients.

circRNA	AUC	95% CI	*P* value	Sensitivity	Specificity
hsa_circ_0082081	0.66	0.522–0.798	0.033	0.833	0.433
hsa_circ_0113854	0.689	0.555–0.823	0.012	0.867	0.5
hsa_circ_0124644	0.872	0.785–0.960	<0.001	0.867	0.767
hsa_circ_0098964	0.82	0.707–0.933	<0.001	0.8	0.867
hsa-circRNA5974-1	0.743	0.619–0.867	0.001	0.633	0.733

The data were evaluated according to the cut-off point.
