# Supplementary material for: Peripheral blood circular RNA hsa_circ_0124644 can be used as a diagnostic biomarker of coronary artery disease
Source: Sci Rep. 2017 Jan 3;7:39918. doi: 10.1038/srep39918 (PMC5206672; doi:10.1038/srep39918)
Supplement: Supplementary Tables [file srep39918-s1.pdf]

Peripheral blood circular RNA hsa_circ_0124644 can be used as a diagnostic biomarker of coronary artery disease

Zhenzhou Zhao[1, &], Xuejie Li[1, &], Chuanyu Gao[1], Dongdong Jian[2], Peiyuan Hao[1], Lixin Rao[1], Muwei Li[1, *]

[1]Department of Cardiology, People's Hospital of Zhengzhou University, Zhengzhou University, Zhengzhou, China.

[2]Department of Cardiology, The First Affiliated Hospital of Zhejiang University, Zhejiang University, Hangzhou, China.

[&]These authors contributed equally to this work.

[*]Correspondence and requests for materials should be addressed to M.W.L. Email: limuwei@medmail.com.cn; Fax: 86-0371-65580358.

	**Control group (n = 12)**	**CAD group (n = 12)**	***P* value**
Male gender	6 (50.0%)	6 (50.0%)	1.0
Age (years)	62.0 ± 5.9	62.4 ± 5.6	0.861
Hypertension	4 (33.3%)	5 (41.7%)	1.0
DM	6 (50.0%)	6 (50.0%)	1.0
Smoker	2 (16.7%)	3 (25.0%)	1.0
HbA1c (%)	6.2 ± 0.9	6.1 ± 0.8	0.775
TC (mmol/L)	4.0 ± 1.0	3.9 ± 1.0	0.839
TG (mmol/L)	1.1 ± 0.5	1.4 ± 0.5	0.190
HDL (mmol/L)	1.2 ± 0.2	1.0 ± 0.2	0.153
LDL (mmol/L)	2.0 ± 0.8	2.0 ± 0.7	0.934
ALT(U/L)	28 (19.5, 54)	29 (25.3, 27.75)	0.840
AST (U/L)	21.7 ± 5.9	21.8 ± 5.3	0.942
Scr (μmol/L)	59.4 ± 7.8	66.2 ± 11.8	0.106
FT4I (pmol/L)	14.1 ± 2.1	14.9 ± 1.2	0.277

Supplementary Table S1

	**Control group (n = 30)**	**CAD group (n = 30)**	***P* value**
Male gender	12 (40%)	17 (56.7%)	0.301
Age (years)	57.3 ± 7.4	59.3 ± 7.1	0.284
Hypertension	10 (33.3%)	16 (53.3%)	0.193
DM	7 (23.3%)	13 (43.3%)	0.171
Smoker	8 (26.7%)	12 (40%)	0.411
HbA1c (%)	5.4 (5.0, 5.7)	5.6 (5.3, 6.7)	0.111
TC (mmol/L)	4.0 ± 0.7	4.4 ± 0.8	0.057
TG (mmol/L)	1.9 ± 0.8	1.7 ± 0.6	0.232
HDL (mmol/L)	1.0 ± 0.2	1.1 ± 0.2	0.091
LDL (mmol/L)	2.2 ± 0.6	2.6 ± 0.8	0.052
ALT (U/L)	22.7 ± 6.0	24.9 ± 9.3	0.297
AST (U/L)	23.1 ± 5.0	21.8 ± 6.2	0.387
Scr (μmol/L)	67.6 ± 13.3	66.8 ± 20.1	0.857
FT4I (pmol/L)	14.6 ± 1.2	15.2 ± 1.5	0.083
CADS	1.8 ± 0.8	27.8 ± 12.6	< 0.001

Supplementary Table S2

	**Control group (n = 115)**	**CAD group (n = 137)**	***P* value**
Male gender	48 (41.7%)	73 (53.3%)	0.068
Age (years)	59.2 ± 8.7	61.4 ± 7.3	0.071
Hypertension	41 (35.7%)	59 (43.1%)	0.231
DM	30 (26.1%)	49 (35.8%)	0.099
Smoker	30 (26.1%)	38 (27.7%)	0.769
HbA1c (%)	6.0 ± 0.9	6.2 ± 0.9	0.105
TC (mmol/L)	3.5 ± 1.3	4.1 ± 1.3	0.001

TG (mmol/L)	1.33 ±0.5	1.91 ±0.5	< 0.001
HDL (mmol/L)	1.4 ±0.2	1.3 ±0.3	< 0.05
LDL (mmol/L)	2.7 ±0.1	3.1 ±0.9	< 0.001
ALT (U/L)	28.2 ±7.3	29.6 ±9.4	0.201
AST (U/L)	28.3 ±7.3	28.9 ±9.2	0.555
Scr (μmol/L)	64.7 ±14.9	63.6 ±13.9	0.548
FT4I (pmol/L)	15.7 ±1.7	16.0 ±2.9	0.267

Supplementary Table S3

Supplementary Tables S1-S3. The clinical and demographic characteristics of CAD patients and control individuals. DM, diabetes mellitus; LDL, low density lipoprotein; TC, total cholesterol; TG, triglycerides; HDL, high density lipoprotein; LDL, low density lipoprotein; ALT, alanine transaminase; AST, aspartate transaminase; Scr, serum creatinine; FT4I, free thyroxine index.

Gene	Forward	Reverse	Product length
hsa_circRNA5974-1	TTCCAAAGGAGTTGCTCAGGAC	GACAGCTGCCTTGACAATGACT	94
hsa_circ_0124644	TGCCTTGGAGTTATGGAGACAGA	TCTGGCCGGTGAGATACAAGT	119
hsa_circ_0113854	AGGTTGGGTGTTGAAAACAAAGAC	TGATTGTGAGGCAGCTCCAGA	169
hsa_circ_0098964	CCCGACAGACCGTCATCCT	TATGACCTTCTCCCAGGGTCC	127
hsa_circ_0082081	CTTTGCTGACCTGTAGCTGGTA	CATTGAGGAAGGCCTGGAACC	80
hGAPDH	TGTTGCCATCAATGACCCCTT	CTCCACGACGTACTCAGCG	202

Supplementary Table S4. Nucleotide sequences of primers used for qPCR.

Upregulated circRNAs	Fold change	*P* value	Downregulated circRNAs	Fold change	*P* value
hsa_circ_0002326	2.438947363	0.035560365	hsa_circ_0001481	2.258483105	0.032302029
hsa_circ_0049995	2.04805298	0.02107932	hsa_circ_0023903	3.76992748	0.001954939
hsa_circ_0062596	2.158444565	0.010621286	hsa_circ_0040707	2.607651871	0.019346175
hsa_circ_0082081	2.083496911	0.006557032	hsa_circ_0073171	2.086622176	0.04827959
hsa_circ_0096621	2.407478647	0.042578987	hsa_circ_0078837	2.004063659	0.004237045
hsa_circ_0098964	2.40156312	3.46027E-05	hsa_circ_0080952	2.014629478	0.010616973
hsa_circ_0105716	2.01770438	0.009477654	hsa_circ_0096650	2.067454217	0.002598809
hsa_circ_0113854	2.188975449	0.001919049	hsa_circ_0096651	2.033421395	0.001006487
hsa_circ_0116488	2.019703222	0.008170498	hsa_circ_0096666	2.396292325	0.000924614
hsa_circ_0124644	2.041067168	0.001544787	hsa_circ_0117264	2.049234665	0.004314649
hsa_circ_0126672	2.000784361	0.017289931			
hsa-circRNA5974-1	2.140819806	0.006673713			

Supplementary Table S5. Differentially expressed circRNAs between controls and CAD patients.
